# Plantar pressure sensors indicate women to have a significantly higher peak pressure on the hallux, toes, forefoot, and medial of the foot compared to men

**DOI:** 10.1186/s13047-020-00410-2

**Published:** 2020-07-01

**Authors:** Tetsuya Yamamoto, Yuichi Hoshino, Noriyuki Kanzaki, Koji Nukuto, Takahiro Yamashita, Kazuyuki Ibaraki, Kouki Nagamune, Kanto Nagai, Daisuke Araki, Takehiko Matsushita, Ryosuke Kuroda

**Affiliations:** 1 Department of Orthopaedic Surgery, Kobe University Graduate School of Medicine, 7-5-1 Kusunoki-cho, Chuo-ku, Kobe, 650-0017 Japan; 2 Human and Artificial Intelligent Systems, University of Fukui Graduate School of Engineering, Fukui, Japan

**Keywords:** Plantar pressure, In-shoe pressure sensor system, Sex difference, Center of pressure

## Abstract

**Background:**

Sex-related differences of plantar pressure distribution during activities should be thoroughly inspected as it can help establish treatment and prevention strategies for foot and ankle problems. In-shoe measurement systems are preferable without space and activity restrictions; however, previously reported systems are still heavy and bulky and induce unnatural movement. Therefore, a slim and light plantar pressure sensor was newly developed to detect the effect of sex difference on plantar pressure during standing and walking.

**Methods:**

One-hundred healthy adult volunteers (50 women and 50 men) were recruited. Ten plantar pressure sensors were implanted in a 1-mm thick insole, with a total weight of 29 g. Plantar pressure was recorded with 200 Hz during 3 s of standing and while walking 10 steps. The maximum loads during standing and walking were analyzed in each sensor, and the results were compared between different areas of the foot in the antero-posterior direction and the medio-lateral direction and between different time points. The movement of the center of pressure (COP) during walking was also evaluated. Analyses were adjusted for body mass index and gait speed.

**Results:**

The movement of COP was constant for both sexes. In all cases, the maximum load was observed on the medial of the foot. Women had a significantly higher peak pressure on the hallux, toes, forefoot, and medial aspect of the foot compared to men while standing and walking (*p* < .05).

**Conclusions:**

A newly introduced in-shoe plantar pressure sensor demonstrated a typical loading transition pattern of the foot. Furthermore, higher plantar pressure in the forefoot was detected in healthy women as compared to men during standing and walking activities.

## Introduction

Analysis of the loading condition of the sole during walking and running is vital to improve footwear design [1], sports performance analysis and injury prevention [2], balance control [3], and for diagnosing diseases [4]. Various foot pressure evaluation devices have been developed, and the currently available systems are categorized into the plantar platform system and the in-shoe system.

At present, over 50 different devices are used for foot loading analysis [5]. Some systems require specialized laboratories, whereas others require relatively expensive and delicate insoles with pressure sensors [6]. The in-shoe system is preferable over the plantar platform system because of its portability, simplicity, and applicability on various shoe types [7], and participants can perform natural movement during the experiment without the restriction of space and activity [8]. However, existing in-shoe systems are heavy and bulky, and induce unnatural movement, urging the need of a light and slim in-shoe type sensor.

Skeletal structure and muscular strength differ between women and men. However, the effect of sex on plantar pressure during activities remains undetermined. Putti et al. did not find any sex-based differences in plantar pressure while walking eight steps on the same walkway [9]. Darlene et al. reported that no significant differences were found in normalized plantar pressure values between men and women [10]. On the other hand, in some investigations, there was a significant difference in the peak pressure between men and women. Demirbuken et al. reported that higher toe peak pressure was identified more often in women than in men during early adolescence [11]. Chung et al. indicated that men had higher peak pressure in the medial toe and all forefoot areas than women [12]. McKay et al. reported that there was no significant difference between boys and girls aged 3–9 years, at ages 10–19, men had significantly higher midfoot peak pressure than women and at age 20 and older men had significantly higher rearfoot peak pressures than women [13].

To examine the effect of sex difference on plantar pressure, testing activities should be performed in positions as natural as possible, without any obstruction from the measurement.

Therefore, a slim plantar pressure sensor was newly developed to measure plantar pressure of healthy adults during unrestricted activities. The purpose of this study was to evaluate pressure distribution on the sole during gait and stance using the newly developed in-shoe measurement system and compare the results between sexes.

## Materials and methods

### Participants

One hundred healthy adult volunteers (50 women and 50 men) were included in the study. Demographic data are shown in Table 1. We analyzed both left and right feet in all 100 cases. There was no difference between left and right feet, and, therefore, the results of the left foot were presented. Participants were excluded if they had experienced foot pain within the previous 6 months, had previously undergone foot surgery, or had presented with congenital or acquired foot deformities on clinical examination. Demographics including age, sex, height, weight, and foot size were recorded. The study protocol was approved by the Institutional Review Board of our institution and written informed consent was obtained from all participants before their enrollment.

**Table 1:**
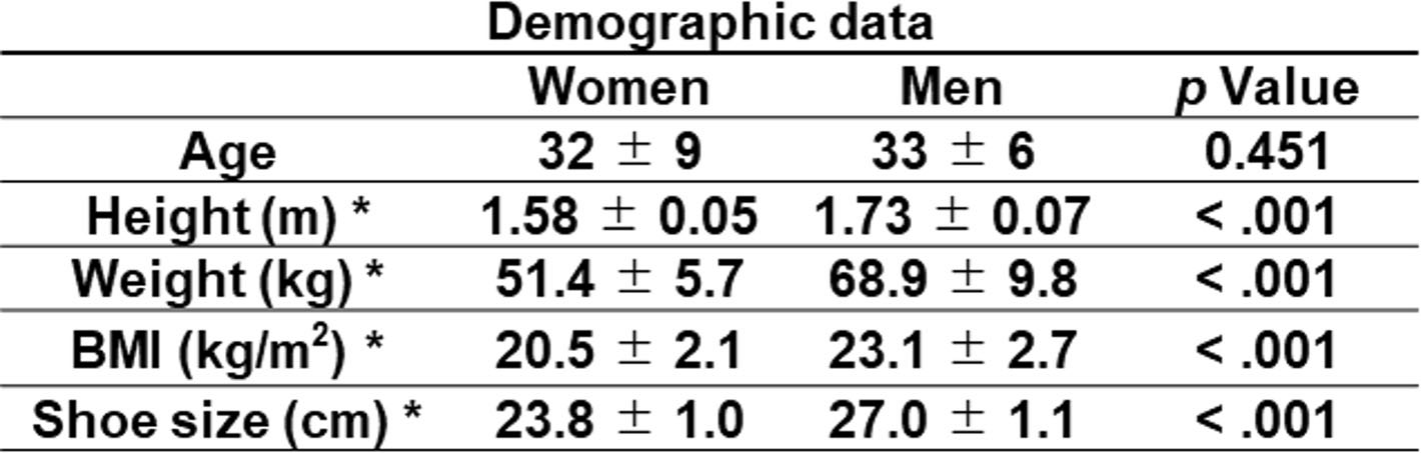
Demographic data.

Sex comparison of demographic data, *p < .05 significantly higher, Paired t-test. Values are given as mean ± SD.

### Measurement devices

**Fig. 1:**
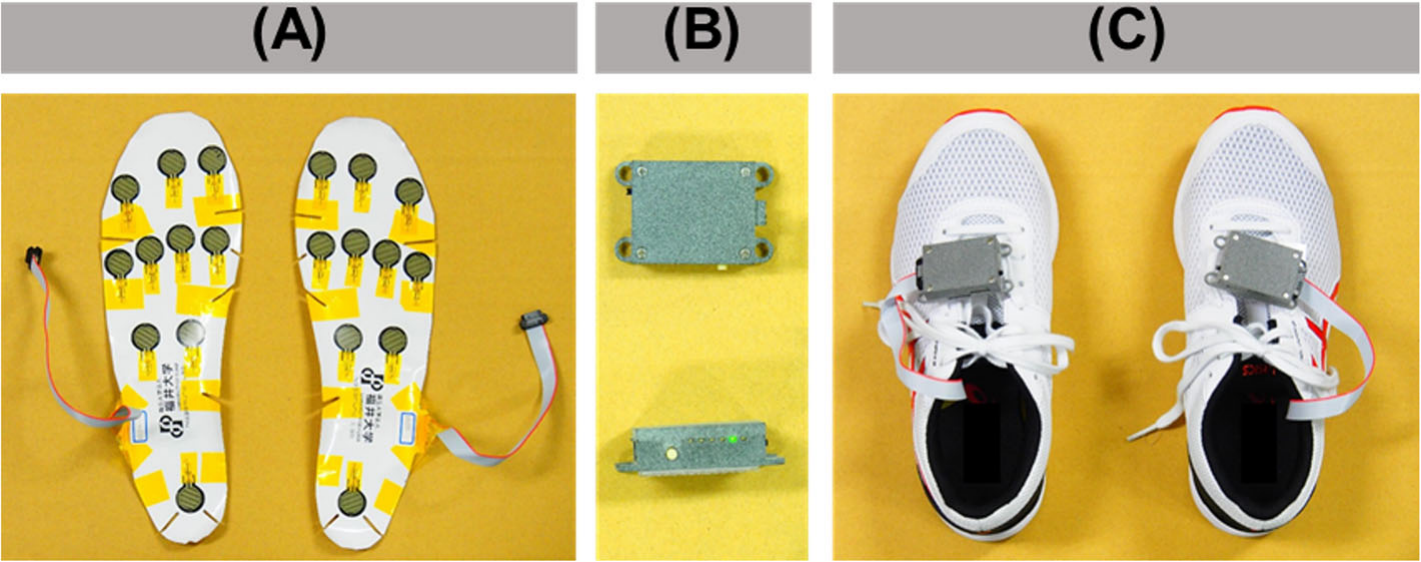
Plantar pressure measurements. **a** Ten of the sensors are attached underneath the insole and connected to the measuring unit. The sensors are only 1 mm in thickness and total 12 g in weight. **b** The measuring unit is only 17 g in weight and collects data at 200 Hz. **c** The sensor is placed underneath the insole and connected to the measuring unit on the top of the foot

The newly developed plantar pressure sensor (University of Fukui Graduate School of Engineering, Japan) has 10 sensors of 1 mm thickness and 12 g weight. They are connected to a measuring unit that has 200 Hz of sampling rate and is 17 g in weight (Fig. 1). Three sensors were placed on the toes, four on the forefoot, two on the midfoot, and one on the hindfoot. Four sizes (23 to 28 cm) of sports shoes and corresponding sizes of plantar pressure sensors were prepared to provide the best fit shoes for each participant.

### Measurement and analysis

**Fig. 2:**
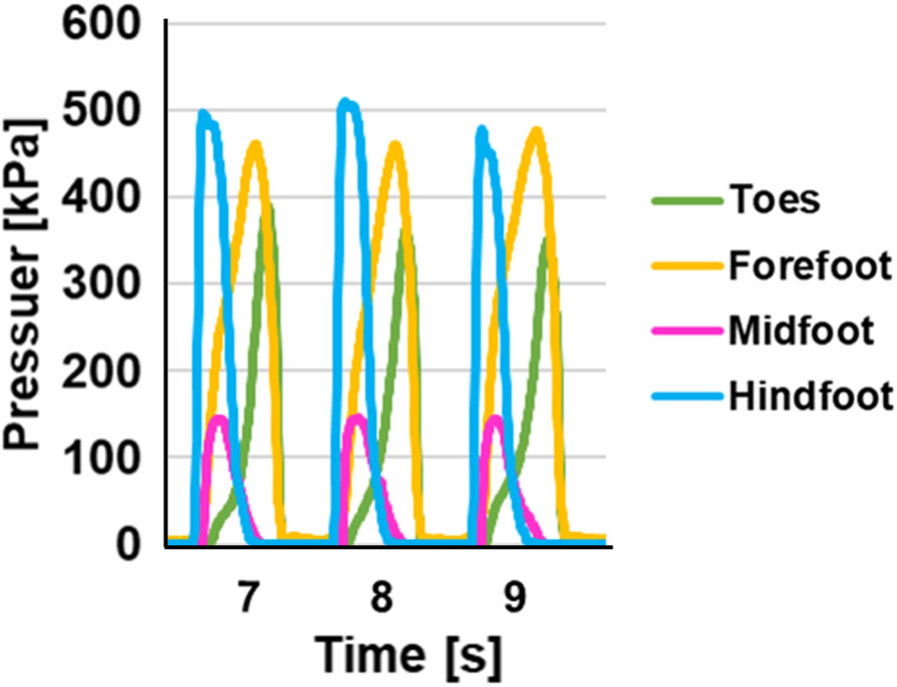
Plantar pressure during walking. Plantar pressure in each the toes, forefoot, midfoot and hindfoot area during walking. The maximum loading in each part reached its peak starting from the hindfoot, the midfoot and the forefoot, consistently

**Fig. 3:**
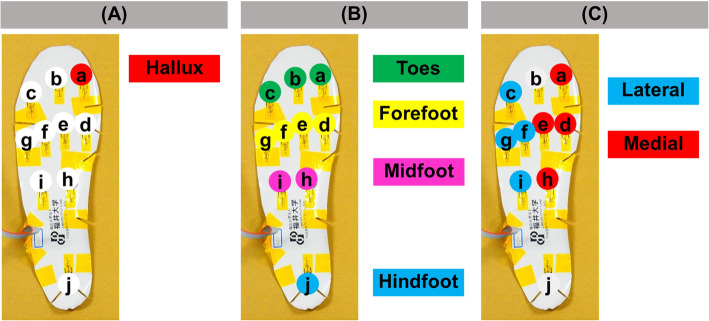
The evaluation of plantar pressure. **a** The value of the sensor a in the figure was defined as Hallux. **b** Areas for analyzing weight-bearing point in anteroposterior direction. The evaluation was divided into four parts: toes, forefoot, midfoot, and hindfoot, and the average value was calculated. **c** Areas for analyzing weight-bearing point in medio-lateral direction. The average value was calculated separately for the medial and lateral

The participants were allowed to acclimatise to the shoes by walking a minimum of 10 steps before data collection, which did not begin until the participant verified that they were comfortable. Each participant rested for 3 s after wearing the device, after which plantar pressure was measured while standing. Next, participants were instructed to walk at least 10 steps at a comfortable speed while the plantar pressure was recorded (Fig. 2). The maximum loading while walking was measured in the hallux (the sensor a), and the movement of the maximum loading point while walking was analyzed by evaluating in different areas of antero-posterior (AP) direction and medio-lateral (ML) direction (Fig. 3). The hallux was measured individually, considering that there might be a difference in the hallux between women and men because hallux valgus was more common in women. The prevalence of hallux valgus was 22.8% in Japan. The prevalence in women was 2.54 times higher than in men [14]. Results were compared between sexes by converting body mass index (BMI) to 22 and gait speed to 118 steps / min (Excel 2016, Microsoft and COP graph creator, University of Fukui Graduate School of Engineering, Japan). The gait speed was 120 ± 9 steps / min for women and 116 ± 11 steps / min for men and there was no significant difference between men and women. Since the overall average was 118 steps / min, we used that value for adjustment. The movement of COP during walking was analyzed in the AP and ML directions. When compared based on sex, the sensor was converted to 25.5 cm equivalent and calculated.

### Statistical analysis

A two-sample t-test was used for a sex-based comparison of the data (Excel 2016, Microsoft). For all analyses, statistical significance was set at *P* < .05. All analyses except of demographic data was used Bonferroni’s correction for multiple comparisons. All data were reported as the mean ± SD.

## Results

**Fig. 4:**
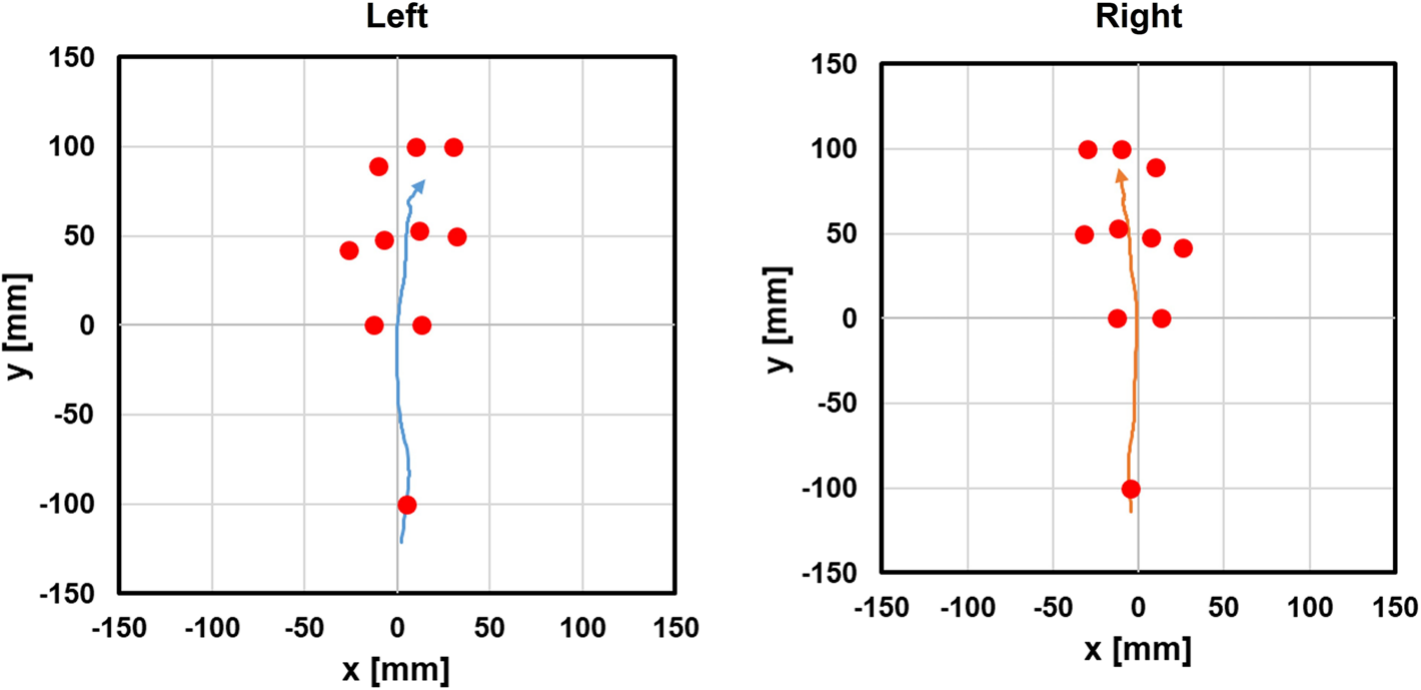
The COP movement. A typical case of COP movement during walking. The COP movements were similar across participants such that it translated from the hindfoot through the middle of midfoot, finally toward the base of first toe

Women had a significantly higher peak pressure on the hallux, toes, forefoot, and medial aspect of the foot compared to that of men during standing (Table 2. All; *p* < .05). Similar to the results during standing, a significantly higher peak pressure was observed on the hallux, toes, forefoot, and medial aspect of the foot in women than men during walking (Table 2, All; *p* < .05). In all cases, greater loading was applied to medial aspect of the foot in the middle of the stance phase than lateral. COP movements were similar across participants such that they translated from the hindfoot through the middle of the midfoot, and finally toward the base of the first toe (Fig. 4). There were no significant differences between sexes (Table 3, All; *p* > .05).

**Table 2:**
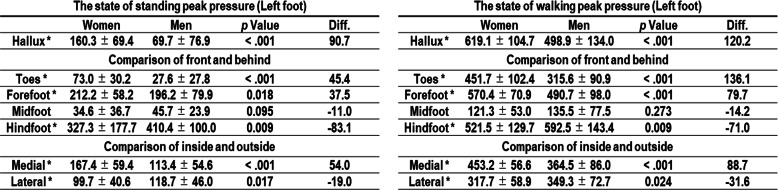
The state of standing and walking peak pressure.

Sex comparison of the state of standing and walking peak pressure, *p < .05 significantly higher, Paired t-test, Bonferroni’s correction. Values are given as mean ± SD (kPa). Diff. = Mean difference in women from men.

**Table 3:**
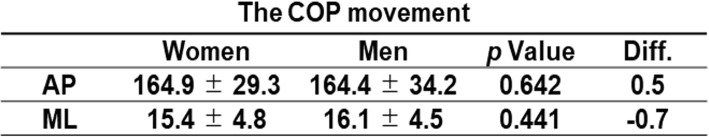
The COP movement.

Sex comparison of the COP movement, there was no significant difference between sex, Paired t-test. Values are given as mean ± SD (mm). Diff. = Mean difference in women from men. *AP* Antero-posterior, *ML* Medio-lateral.

## Discussion

The most important finding of this study was that women applied a significantly higher peak pressure on the hallux, toes, forefoot, and medial aspect of the foot while both standing and walking than men. Women have a higher pelvic tilt and a center of gravity anterior to men [15], which can result in higher plantar pressure at the toes and forefoot. The movement of knee-in while walking was likely to occur in women because of weaker gluteus medius strength compared to men [16]. Thus, the peak pressure at the medial aspect of the foot may be higher. In previous reports, the results regarding the difference in peak pressure between men and women were varied. Some reports indicated that there was no significant difference between men and women [9, 10], and some reports indicated that men have higher peak pressure in the medial toe and all forefoot areas than women [12]. On the other hand, higher toe peak pressure was identified more often in women than in men during early adolescence [11]. It is also reported that the peak pressure shifts from the hind foot to the forefoot depending on the age [13].

Technical improvement of the measurement system was demonstrated for evaluation of the plantar pressure during natural activities. The Pedar system (Novel gmbh, Munich, Germany) and F-scan system (Tekscan, Inc., Boston, MA) are the main models of the in-shoe plantar pressure measurement system in the previous studies [17, 18]. However, they are relatively heavy (The Pedar system; 400 g, F-scan system; 400 g) and bulky (The Pedar system; 600 cm^3^, F-scan system; 300 cm^3^). To evaluate natural walking, the size and weight of a measurement device should be as light and small as possible. The newly developed in-shoe device in the current study weighs 17 g and has a volume of 15 cm^3^, making it possible to perform standing and walking with less interference than previous products. Additionally, conventional products have 50 Hz of sampling rate, whereas this device has 200 Hz of sampling rate. Therefore, the new system could have less chance to miss some instant and important changes of the plantar pressure during activities. The newly introduced device can be applied to faster movements, such as running and sports activities.

The COP is defined as the centroid of the total number of active sensors, which suggests the spatial distribution of pressure over time [19]. It has been suggested that the COP provides greater insight into dynamic foot function compared to pressure at discrete regions [20]. Buldt et al. reported a difference between the planus and a normal foot in relation to the medial shift of the lateral-medial force index during terminal stance [21]. In this study, COP movements were similar across healthy adult participants such that they translated from the hindfoot through the middle of the midfoot, and finally toward the base of the first toe. Future studies comparing healthy feet with pathological conditions, such as flat foot and hallux valgus, are warranted.

Although this study did not include cases of hallux valgus (HV), women had a higher plantar pressure at the hallux than men. Nix reported a meta-analysis that estimated that female HV prevalence (30%) was 2.3 times greater than in men (13%) [22]. Studies have reported plantar pressure in HV in the past; however, their results have been inconsistent. Some have reported a high plantar pressure on the hallux [23, 24], while others have reported an inverse correlation between severity and plantar pressure at the hallux [25]. There are various causes of HV; however, this sex-based difference in pressure can be suggested as one of the causes.

The findings of this study should be considered after taking into consideration five limitations. We have not recorded the foot posture of the participant in this study, however, according to a systematic review of foot posture by Buldt et al., there was a significant difference in peak pressure between the pes cavus, the pes planus, and the normal foot [6]. Since we selected healthy foot participants, it was considered that foot posture had little effect on peak pressure. We have not recorded the foot morphology of the participant in this study. Mahshid et al. reported that Japanese women had narrower feet in the heel and forefoot, and their instep, first and fifth toes and navicular height were also lower than men’s [26]. Zhao et al. demonstrated that Japanese men generally had longer, larger and higher arched feet than women [27]. Since we adjusted the shoe size to 25.5 cm and compared, it was considered that foot morphology had little effect on peak pressure. Plantar pressure measurement systems are limited in that they only measure force perpendicular to the sensor surface. Therefore, other relevant forces including shear force cannot be measured. However, the current study examined the force during standing and walking when a vast majority of force is applied perpendicular to the foot. The influence of other related forces might be considered when more active sporting activities are involved, i.e. turning, stop-and-go motions. Footwear characteristics such as sole bending stiffness are likely to influence the parameters. To avoid this impact, the same shoes of different sizes were used in the current study; however, care should be taken when using different shoes in future studies. Lastly, although statistical significance was achieved in the comparisons, the sample size might be insufficient to apply this study result to the general population of wider age and/or other cultural backgrounds. It should be noted that the current study results stem from participants who were relatively younger than most foot and ankle patients, but older than most active sport athletes.

The current study result could be used to develop sex specific design of insole or shoes. Wearing appropriate shoes may help prevent foot and ankle pathological conditions. In addition, checking the COP movement may help improve gait balance and gait posture. In order to invent therapeutic ones, further detailed evaluations of plantar pressure in pathological feet are needed, and the new measurement tool might be applicable.

## Conclusions

Women tend to put more pressure on the front and medial side of the foot during natural standing and walking than men.

## Data Availability

The datasets used and/or analyzed during the current study are available from the corresponding authors on reasonable request. Please contact authors for data requests (MD, PhD. Yuichi Hoshino – email address: yuichi-h@mta.biglobe.ne.jp).

## Abbreviations

COP: Center of pressure
AP: Antero-posterior
ML: Medio-lateral
HV: Hallux valgus
